# Nod-Like Receptor Protein-3 Inflammasome Plays an Important Role during Early Stages of Wound Healing

**DOI:** 10.1371/journal.pone.0119106

**Published:** 2015-03-20

**Authors:** Eileen M. Weinheimer-Haus, Rita E. Mirza, Timothy J. Koh

**Affiliations:** 1 Department of Kinesiology and Nutrition, University of Illinois at Chicago, Chicago, Illinois, United States of America; 2 Center for Wound Healing and Tissue Regeneration, University of Illinois at Chicago, Chicago, Illinois, United States of America; Institute of Basic Medical Sciences, Chinese Academy of Medical Sciences, CHINA

## Abstract

The Nod-like receptor protein (NLRP)-3 inflammasome/IL-1β pathway is involved in the pathogenesis of various inflammatory skin diseases, but its biological role in wound healing remains to be elucidated. Since inflammation is typically thought to impede healing, we hypothesized that loss of NLRP-3 activity would result in a downregulated inflammatory response and accelerated wound healing. NLRP-3 null mice, caspase-1 null mice and C57Bl/6 wild type control mice (WT) received four 8 mm excisional cutaneous wounds; inflammation and healing were assessed during the early stage of wound healing. Consistent with our hypothesis, wounds from NLRP-3 null and caspase-1 null mice contained lower levels of the pro-inflammatory cytokines IL-1β and TNF-α compared to WT mice and had reduced neutrophil and macrophage accumulation. Contrary to our hypothesis, re-epithelialization, granulation tissue formation, and angiogenesis were delayed in NLRP-3 null mice and caspase-1 null mice compared to WT mice, indicating that NLRP-3 signaling is important for early events in wound healing. Topical treatment of excisional wounds with recombinant IL-1β partially restored granulation tissue formation in wounds of NLRP-3 null mice, confirming the importance of NLRP-3-dependent IL-1β production during early wound healing. Despite the improvement in healing, angiogenesis and levels of the pro-angiogenic growth factor VEGF were further reduced in IL-1β treated wounds, suggesting that IL-1β has a negative effect on angiogenesis and that NLRP-3 promotes angiogenesis in an IL-1β-independent manner. These findings indicate that the NLRP-3 inflammasome contributes to the early inflammatory phase following skin wounding and is important for efficient healing.

## Introduction

Normal wound healing consists of overlapping phases of hemostasis, inflammation, tissue formation, and remodeling. During the inflammatory phase, leukocytes infiltrate the wound site to eliminate microbes and clear the wound of damaged tissue [1]. These cells also provide growth factors and cytokines that have profound effects on subsequent tissue formation and angiogenesis [2–5]. As such, the inflammatory response influences each subsequent phase of healing and is thought to be essential in re-establishing cutaneous homeostasis following injury. However, excessive or prolonged inflammation is a hallmark of chronic wounds [6], is thought to contribute to impaired healing in diabetes [7–11], and has been linked to increased scarring [12,13].

Interleukin (IL)-1 is a pleiotropic pro-inflammatory cytokine that is produced by various cells such as neutrophils, macrophages, fibroblasts and keratinocytes [14,15]. Activity of both IL-1α and IL-1β is mediated by the IL-1 receptor (IL-1R) and inhibited by the IL-1 receptor antagonist (IL-1Ra) [16]. Interestingly, wounds from IL-1R knockout mice showed reduced scarring and inflammatory cell accumulation [17], whereas IL-1Ra knockout mice experienced impaired wound healing accompanied by an exaggerated inflammatory cell infiltration [18]. In addition, elevated levels of IL-1β have been found in wounds from diabetic humans and mice, which exhibit a persistent inflammatory response and impaired healing [9,10,19,20]. Collectively, these findings suggest that the IL-1 pathway plays a central role in the inflammatory response during wound healing and that elevated levels of IL-1 may contribute to impaired healing.

Following tissue injury, a variety of pro-inflammatory danger signals are thought to induce the assembly and activation of a multiprotein complex called the Nod-like receptor protein (NLRP)-3 inflammasome [21–23]. During activation, procaspase-1 is recruited to the NLRP-3 complex and cleaved to produce active caspase-1, which in turn cleaves proIL-1β to produce the active cytokine. Inflammasome components can be expressed in various cell types involved in wound healing including macrophages and keratinocytes [24–26]. Furthermore, the inflammasome/IL-1β pathway is involved in the pathogenesis of various inflammatory skin diseases [27–29], and we and others have previously shown that sustained NLRP-3 inflammasome activity contributes to impaired healing in diabetic wounds [25,30]. However, little is known about the role of the NLRP-3 inflammasome in normal skin wound healing. Thus, we investigated the healing response in mice lacking components of the NLRP-3 inflammasome following cutaneous wounding. We hypothesized that mice deficient in either NLRP-3 or caspase-1 would have reduced IL-1β production, and thus, a downregulated inflammatory response and accelerated wound healing.

## Materials and Methods

### Animals

C57Bl/6 wild-type (WT) controls were obtained from Jackson Laboratories. Breeding pairs of NLRP-3 knockout (KO) mice on a C57Bl/6 background were provided by Genentech and caspase-1 KO mice on a C57Bl/6 background were provided by Drs. Mihai Netea and Leo Joosten, Radboud University Nijmegen Medical Center. Experiments were performed on 12–16 week-old mice.

### Ethics statement

All procedures involving animals were approved by the Animal Care Committee at the University of Illinois at Chicago (protocol #12–207). All animals were housed under standard conditions and treated according to the Guide for the Care and Use of Laboratory Animals of the NIH.

### Excisional wounding and treatment

Mice were subjected to full-thickness excisional wounding on the skin of their dorsum with an 8 mm biopsy punch as described previously [7,20]. As indicated, wounds of NLRP-3 KO mice were topically treated with either 300 pg/wound or 3 μg/wound of recombinant mouse IL-1β (PeproTech, Rocky Hill, New Jersey, USA) in F-127 pluronic gel (50 μl of a 25% gel in saline) [31,32] or a vehicle control (PBS-loaded gel) on days 1, 2 and 3 post-injury. In addition, wounds from WT mice were treated with the caspase-1 inhibitor Ac-YVAD-cmk (20 μmol/L; Cayman Chemical) in F-127 pluronic gel (50 μl of a 25% gel in saline) or a vehicle control (DMSO-loaded gel) immediately post-injury and on day 2 post-injury. Wounds were harvested at day 5 post-wounding.

### Wound healing assays

Wound healing was assessed using our previously published assays of re-epithelialization, granulation tissue formation, collagen deposition, and angiogenesis using hematoxylin and eosin, Masson’s Trichrome and CD31 stained cryosections [2,7,33]. Two wounds per mouse were collected and sectioned from one edge to well past the center. Sections were then selected from the center of the wound by microscopic assessment. Three 10-μm sections judged to be at the actual center of the wound were used for re-epithelialization and granulation tissue thickness measurements. Adjacent three 10-μm sections were used for trichrome staining, CD31 staining, and inflammatory cell staining (described below). For all assays, digital images were obtained using a Nikon Instruments 80i microscope and DS-QI1 digital camera and analyzed using NIS Elements image analysis software (Nikon, Melville, NY, USA).

### Re-epithelialization and granulation tissue thickness

Wound re-epithelialization was measured by morphometric analysis of wound sections. Sections taken from the center of the wound were stained with H&E and the distance between the wound edges, defined by the distance between the first hair follicle encountered at each end of the wound, and the distance that the epithelium had traversed into the wound, were measured using image analysis software. The percentage of re-epithelialization [(distance traversed by epithelium)/(distance between wound edges) ×100] and granulation tissue thickness [(area of granulation tissue present)/(distance between wound edges)] was calculated for three sections per wound and was averaged over sections to provide a representative value for each wound.

### Collagen Deposition and Angiogenesis

Dermal healing was assessed using Masson's trichrome stain for collagen deposition and immunohistochemical staining for CD31 for angiogenesis. For trichrome analysis, staining was performed according to the manufacturer's directions (IMEB, San Marcos, CA, USA), and image analysis software (NIS Elements) was used to quantify the percentage of blue collagen-stained area relative to the total area of the wound bed. For angiogenesis, an antibody against CD31 (BD Pharmingen, San Diego, CA, USA) was used in conjunction with procedures identical to those for inflammatory cells described below, and image analysis software was used to quantify the percentage of CD31-stained area relative to the total area of the wound bed. For each assay, digital images covering the majority of the wound bed (usually three images at ×20 magnification) were first obtained. The percent area stained in each image was then quantified by counting the number of pixels staining above a threshold intensity and normalizing to the total number of pixels. Threshold intensity was set such that only clearly stained pixels were counted. The software allowed the observer to exclude staining identified as artifact, large vessels, and areas deemed to be outside the wound bed. For both trichrome and CD31 staining, three sections per wound were analyzed, and data were averaged over sections to provide a representative value for each wound.

### Inflammatory Cell Accumulation

Immunohistochemical analysis was performed on cryosections taken from the center of each wound [2,7,33]. Sections were air-dried, fixed in cold acetone, washed with PBS, quenched with 0.3% hydrogen peroxide, and washed with PBS. Sections were blocked with buffer containing 3% bovine serum albumin and then incubated with F4/80 antibody to label macrophages (1:100, eBioscience, San Diego, CA, USA) or Ly6G antibody to label neutrophils (1:100, BD Pharmingen, San Diego, CA, USA). Sections were then washed with PBS and incubated with biotinylated anti-rat secondary antibody (1:200, Vector Laboratories, Burlingame, CA, USA). After a wash with PBS, sections were incubated with avidin D-horseradish peroxidase (1:1000) and developed with a 3-amino-9-ethylcarbazole kit (Vector Laboratories). Digital images were obtained using a Nikon Instruments Eclipse 80i microscope with a 20x/0.75 objective, a DS-Fi1 digital camera, and NIS Elements software. The percent area stained in each image was then quantified as described above for trichrome and CD31.

### ELISA

Wounds were homogenized in CelLytic MT Cell Lysis Reagent (10 μl of reagent per mg wound tissue; Sigma Aldrich, St. Louis, MO, USA) supplemented with protease inhibitor cocktail (Sigma Aldrich) using a dounce homogenizer and then centrifuged. Supernatants were used for enzyme-linked immunoassay of IL-1β and TNF-α (eBioscience, San Diego, CA, USA) and VEGF and FGF-2 (R&D Systems, Minneapolis, MN, USA).

### Statistics

Values are reported as means ± standard error. Measurements of wound healing and protein levels were compared between mouse strains using a t-test and between treatments using ANOVA. The Holm-Sidak post hoc test was used when ANOVAs demonstrated significance. Differences between groups were considered significant if *P ≤* 0.05.

## Results

### Reduced inflammation in NLRP-3 KO and caspase-1 KO mice

**Fig 1:**
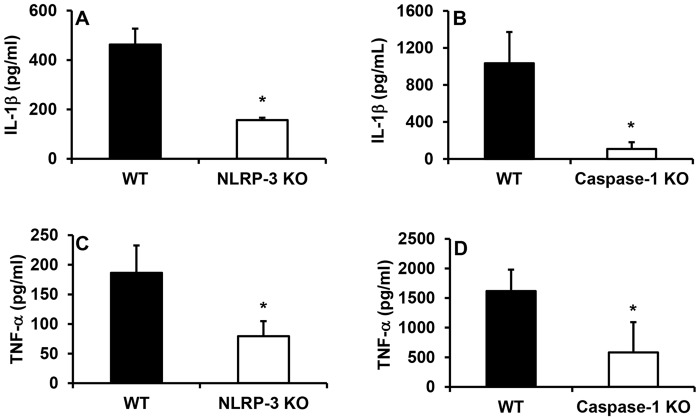
Reduced pro-inflammatory cytokines in wounds from NLRP-3 KO and caspase-1 KO mice. Wounds were harvested on day 5 following injury and levels of (A-B) IL-1β and (C-D) TNF-α were measured in wound homogenates using ELISA. Data presented as mean ± SE, *n* = 4–8 wounds per group. *P≤ 0.05.

**Fig 2:**
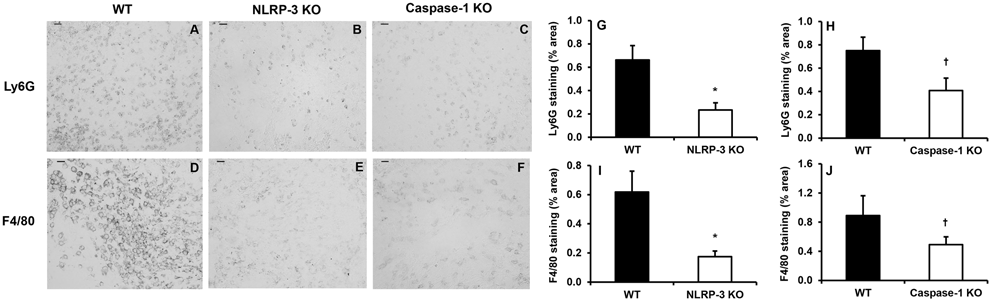
Reduced inflammatory cell accumulation in wounds from NLRP-3 KO and caspase-1 KO mice. Wounds were harvested on day 5 post-injury and cryosections from the center of each wound were stained with antibodies against Ly6G (neutrophils) and F4/80 (macrophages). (A-C) Representative images of Ly6G and (D-F) F4/80 stained sections, scale bar = 0.5 mm. (G-H) Ly6G and (I-J) F4/80 staining was quantified using the percentage of wound bed area stained. For each assay, digital images covering the majority of the wound bed (usually three images at ×20 magnification) were first obtained. The percent area stained in each image was then quantified by counting the number of pixels staining above a threshold intensity and normalizing to the total number of pixels. Data presented as mean ± SE, *n* = 7–8 wounds per group. *P≤ 0.05. ^**†**^ *P* = 0.057 for Ly6G staining (H) and *P* = 0.194 for F4/80 staining (J).

As expected, mice deficient in NLRP-3 or caspase-1 exhibited a reduced inflammatory response at day 5 following wounding compared to WT controls. Wounds in NLRP3-KO and caspase-1 KO mice contained reduced levels of the pro-inflammatory cytokines IL-1β and TNF-α compared to WT controls (Fig. 1A-D). In addition, wound cryosections stained with Ly6G revealed a reduction in neutrophil accumulation in NLRP-3 KO mice versus WT mice and a similar trend in caspase-1 KO mice (Fig. 2A-C,G-H). Furthermore, macrophage accumulation assessed using F4/80 staining was attenuated in NLRP-3 KO mice and showed a similar trend in caspase-1 KO mice (Fig. 2D-F,I-J). These findings suggest that reduced activity of the NLRP-3 inflammasome, and thus lower production of IL-1β, leads to an attenuated inflammatory response in wounds.

### Delayed healing in NLRP-3 KO and caspase-1 KO mice

**Fig 3:**
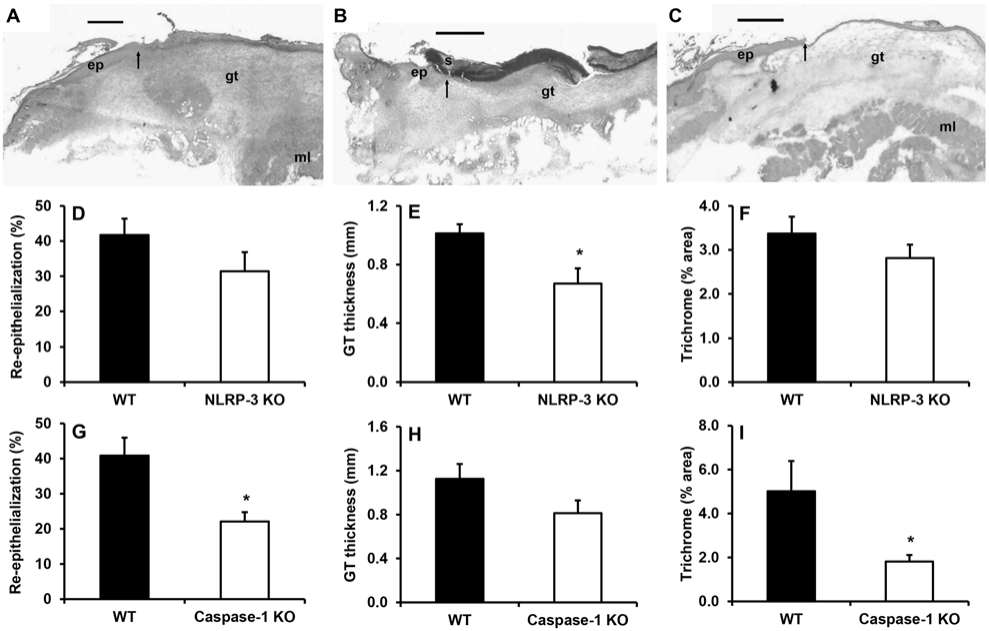
Delayed healing in NLRP-3 KO and caspase-1 KO mice. Images of day 5 wound cryosections stained with H&E for (A) wild-type, (B) NLRP-3 knockout and (C) caspase-1 knockout mice. Approximately one-half of the wound is shown. Note the reduced re-epithelialization and granulation tissue in the NLRP-3 knockout and caspase-1 knockout mice. Scale bar = 0.5 mm. Arrows indicate ends of migrating epithelial tongues. gt, granulation tissue; s, scab; ml, deep muscle. (D,G) Re-epithelialization was measured as the distance traversed by the epithelium divided by the distance between wound edges multiplied by 100 in H&E stained sections of the wound center. (E,H) Granulation tissue thickness was measured as the area of granulation tissue divided by the distance between wound edges in H&E stained sections of the wound center. (F,I) Trichrome staining was measured as percent area stained blue for collagen. Data presented as mean ± SE, *n* = 5–8 wounds per group. *P≤ 0.05.

Associated with the reduced inflammatory response and contrary to our hypothesis, wound healing was delayed in NLRP-3 KO mice and caspase-1 KO mice compared to WT mice. Histological measurements indicated that wounds from caspase-1 KO mice were only 20% re-epithelialized, at day 5 compared to 40% in WT controls; re-epithelialization showed a similar trend in NLRP-3 KO mice (Fig. 3A-D, G). To corroborate these findings, wounds from WT mice were treated with the caspase-1 inhibitor Tyr-Val-Ala-Asp (YVAD). Compared to vehicle control-treated wounds, YVAD treatment resulted in delayed re-epithelialization (Control: 47 ± 3%; YVAD: 34 ± 4%; *P≤* 0.05).

Granulation tissue formation was also decreased in NLRP-3 KO mice (Fig. 3A-C, E, H) and collagen deposition, assessed with trichrome staining, was lower in caspase-1 KO mice (Fig. 3F,I); in each case the KO strain for the other inflammasome component showed a similar but non-significant trend. By day 10 post-injury, the differences in wound healing parameters had disappeared (data not shown), suggesting that loss of NLRP-3 activity and the resulting dampened early inflammatory response induces impaired early healing responses.

### Impaired angiogenesis in wounds from NLRP-3 KO and caspase-1 KO mice

**Fig 4:**
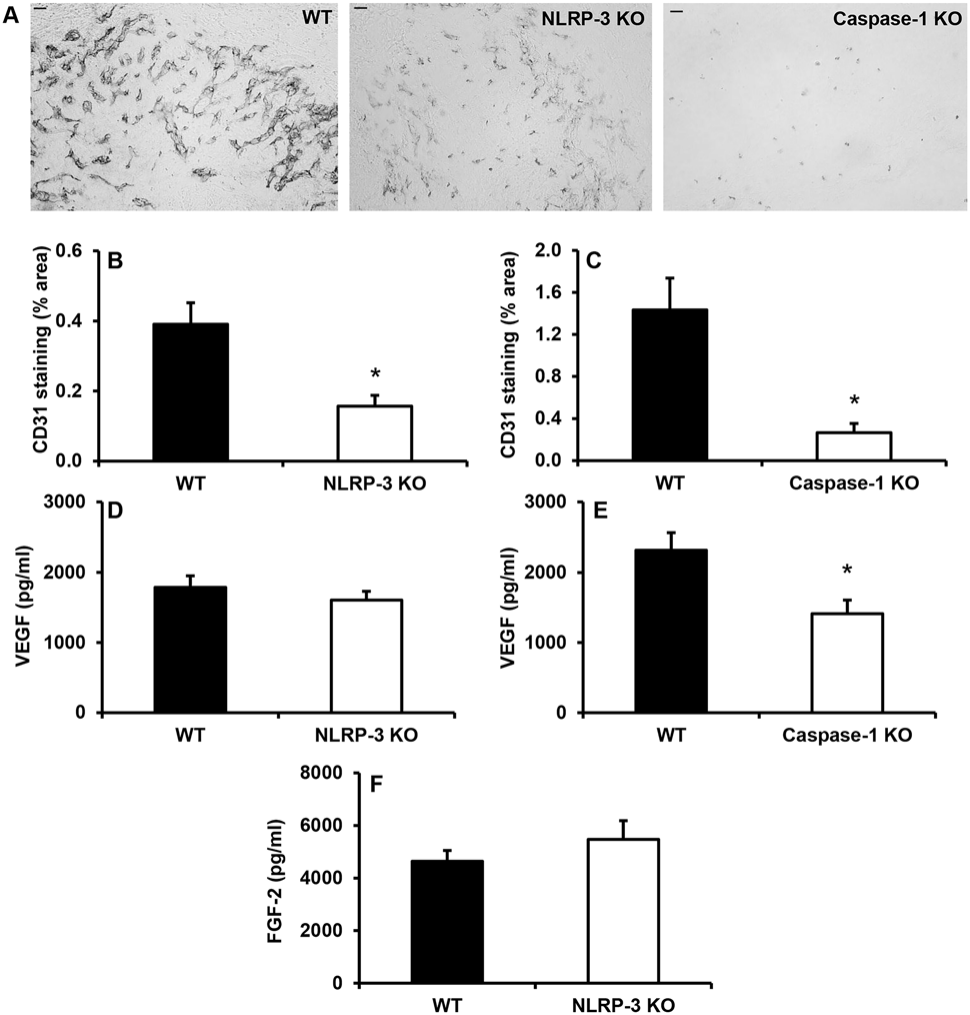
Wounds from NLRP-3 KO and caspase-1 KO mice exhibit impaired angiogenesis. Wound sections were stained with antibodies against CD31. (A) Representative images of CD31 stained sections, scale bar = 0.5 mm. (B-C) CD31 staining was quantified using the percentage of wound bed area stained. (D-E) In addition, levels of the pro-angiogenic growth factors VEGF and (F) FGF-2 were measured using ELISA in wound homogenates. Data presented as mean ± SE, *n* = 5–8 wounds per group. *P≤ 0.05.

Compared to WT controls, wounds from both NLRP-3 KO and caspase-1 KO mice exhibited decreased angiogenesis as assessed by CD31 staining (Fig. 4A-C). In addition, expression of the potent pro-angiogenic growth factor VEGF was reduced in wounds from caspase-1 KO mice versus controls (Fig. 4D-E). However, levels of another pro-angiogenic growth factor, FGF-2, whose release from cells may be influenced by NLRP-3 [34], were not different between NLRP-3 KO mice vs. WT mice (Fig. 4F). Collectively, the reduced inflammatory response and impaired angiogenesis in NLRP-3 KO and caspase-1 KO mice suggests that the NLRP-3-mediated inflammatory response following injury may be necessary for angiogenesis during wound healing.

### IL-1β treatment partially rescues delayed healing in NLRP-3 KO mice

**Fig 5:**
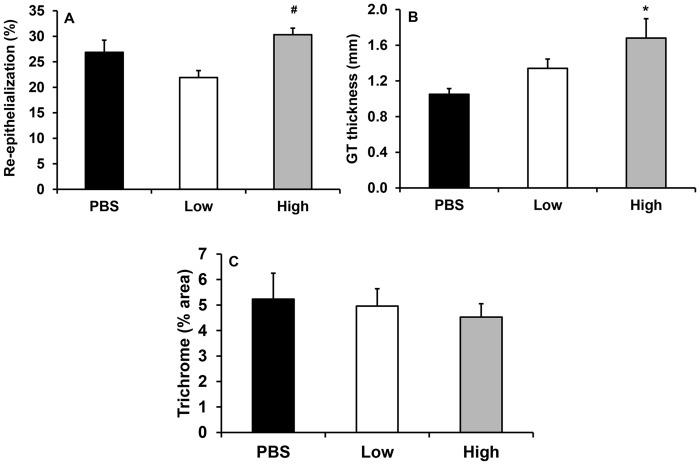
Delayed healing in NLRP-3 knockout mice partially rescued with IL-1β treatment. Wounds of NLRP-3 knockout mice were topically treated with vehicle (PBS) or 300 pg/wound or 3 μg/wound of recombinant IL-1β on days 1, 2 and 3 post-injury. Quantification of (A) re-epithelialization and (B) granulation tissue thickness measured in H&E stained cryosections and (C) trichrome staining measured as percent area stained blue for collagen. Data presented as mean ± SE, *n* = 7–8 wounds per group. *P≤ 0.05 vs. PBS. ^#^ *P≤* 0.05 vs. low.

To determine whether loss of IL-1β in wounds of NLRP-3 KO mice was responsible for the defects observed in wound healing, we performed rescue experiments in NLRP-3 KO mice. Wounds from NLRP-3 KO mice were treated with a physiological or supraphysiological dose (low, 300 pg/wound or high, 3 μg/wound, respectively) of recombinant IL-1β or vehicle (PBS) in attempt to rescue the impaired healing phenotype. Histological assessments of wound cryosections revealed that wounds treated with the higher dose of IL-1β exhibited a trend of accelerated re-epithelialization compared to PBS- (*P* = 0.162) and low dose IL-1β-treated wounds (Fig. 5A). In addition, granulation tissue formation was enhanced in high dose-treated wounds vs. PBS-treated wounds, but collagen deposition was unchanged (Fig. 5B-C). While the percent collagen staining in wounds was not different among treatments, the increased amount of granulation tissue formation in the high dose IL-1β-treated wounds implies an increase in total collagen deposition.

### IL-1β treatment fails to rescue impaired angiogenesis in NLRP-3 KO mice

**Fig 6:**
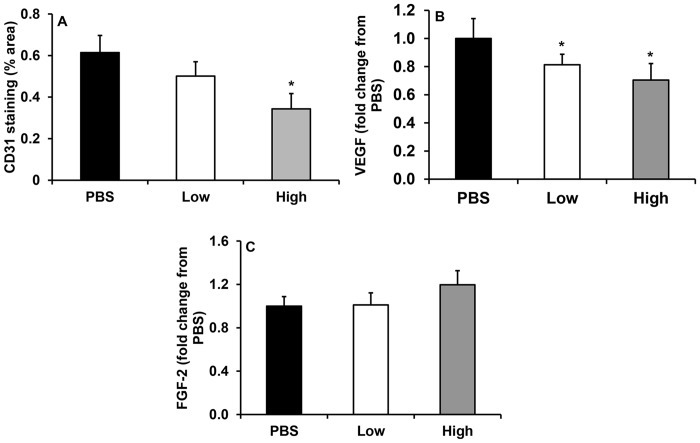
IL-1β treatment unsuccessful in rescuing impaired angiogenesis in NLRP-3 KO mice. Wounds of NLRP-3 knockout mice were topically treated with vehicle (PBS) or 300 pg/wound or 3 μg/wound of recombinant IL-1β on days 1, 2 and 3 post-injury. (A) CD31 staining measured as percent area stained for this endothelial cell marker. Wounds were also homogenized and levels of (B) VEGF and (C) FGF-2 were measured using ELISA. Data presented as mean ± SE, *n* = 6–8 wounds per group. *P 0.05 vs. PBS.

In addition to its pro-inflammatory effects, IL-1β has pro-angiogenic properties [35,36]. Thus, treating wounds of NLRP-3 KO mice with IL-1β may be expected to restore the angiogenic response. However, CD31 staining revealed an unexpected reduction in angiogenesis with high dose IL-1β treatment compared to vehicle-treated wounds (Fig. 6A). Levels of the pro-angiogenic growth factor VEGF were also reduced in both low and high dose IL-1β-treated wounds vs. PBS-treated wounds (Fig. 6B), while FGF-2 levels were unchanged (Fig. 6C). These observations indicate that the improvements in wound healing with IL-1β treatment occur independent of angiogenesis. When considered together with the impaired angiogenesis in NLRP-3 and caspase-1 KO mice, these data indicate that NLRP-3 and caspase-1 may promote angiogenesis through an IL-1β-independent mechanism.

## Discussion

The NLRP-3 inflammasome is a cytosolic, multiprotein complex that assembles in response to various danger signals released following tissue injury [21–23]. Recognition of these stimuli by the inflammasome results in activation of caspase-1, which is required for IL-1β activation and secretion. Although aberrant activity of the NLRP-3 inflammasome and IL-1β has been implicated in the pathophysiology of various inflammatory skin diseases [27–29] and has been shown to contribute to impaired healing in diabetic wounds [20,25,30], less is known regarding the role of the NLRP-3 inflammasome during normal skin wound healing. In the present study, we demonstrate that the NLRP-3 inflammasome plays an important role during the early stages of wound healing. Mice deficient in NLRP-3 or caspase-1 experienced delayed wound healing associated with reduced levels of IL-1β, a dampened inflammatory response, delayed re-epithelialization and granulation tissue formation, and impaired angiogenesis. The impaired healing phenotype in NLRP-3 KO mice was partially rescued by topically treating excisional wounds with IL-1β. These findings ultimately suggest that the early inflammatory response is, at least partly, mediated by the NLRP-3/IL-1β pathway and is important for efficient tissue repair.

Findings from previous studies have indicated that elevated levels of IL-1β and its pro-inflammatory actions impair skin wound healing. Wounds from IL-1Ra knockout mice had increased levels of the chemokines keratinocyte-derived chemokine and macrophage inhibitory protein-1α and accumulation of neutrophils, which was associated with higher IL-1β levels and impaired healing [18]. In mice lacking the IL-1 receptor, inflammatory cell accumulation was reduced, but closure was not altered [17,37]. Elevated levels of IL-1β have also been found in diabetic wounds, which exhibit a persistent inflammatory response and impaired healing [9,10,19,20]. We recently reported that sustained IL-1β expression in wounds from diabetic mice and humans is associated with a pro-inflammatory macrophage phenotype [20]. Importantly, inhibition of the IL-1β pathway in wounds of diabetic mice induced the switch from a pro-inflammatory to a healing-associated macrophage phenotype and improved healing of these wounds. In contrast, NLRP-3 KO and caspase-1 KO mice in the current study experienced impaired healing despite a reduction in inflammatory cell accumulation and IL-1β levels, suggesting that the IL-1β-mediated early inflammatory response is important for efficient repair in non-diabetic mice.

Interleukin-1β is generally considered a pro-angiogenic factor as demonstrated by its role in tumor angiogenesis [36]. IL-1β is thought to function directly by increasing expression of pro-angiogenic factors such as VEGF and FGF-2 in endothelial cells, or indirectly through the activation of infiltrating myeloid cells to produce a variety of cytokines/chemokines, which further activate tissue resident endothelial cells to produce pro-angiogenic factors [35,38]. Indeed, cutaneous wounds from NLRP-3 KO and caspase-1 KO mice had reduced angiogenesis and levels of VEGF, consistent with a previous report [34]. Unexpectedly, angiogenesis and VEGF levels were further reduced in NLRP-3 KO mice that were treated with recombinant IL-1β, suggesting that the NLRP-3/caspase-1 pathway mediated angiogenesis via an IL-1β-independent mechanism.

In addition to IL-1β, the NLRP-3 inflammasome processes and activates proIL-18 and proIL-33 via caspase-1, although the latter remains controversial [39,40]. Few studies have investigated the role of IL-18 or IL-33 in wound healing. In mice, IL-18 protein levels rapidly increased following cutaneous wounding [41]. *In vitro* studies have demonstrated an increased production of extracellular matrix components with IL-18 treatment in cardiac fibroblasts [42]; while speculative, this may suggest a role of IL-18 in granulation tissue formation. In the eye, IL-18 is currently being investigated as a potential therapeutic strategy for the treatment of age-related macular degeneration due to its strong anti-angiogenic actions [43]. Although IL-18 was not measured in the current study, previous studies have reported a defect in the production of mature IL-18 in caspase-1 KO mice [44,45]. Because IL-18 is thought to be anti-angiogenic, we can speculate that the impaired angiogenesis in the NLRP-3 KO and caspase-1 KO mice was likely not mediated by a lack of IL-18.

Following cutaneous wounding, both mRNA and protein expression of IL-33 are elevated in mice [46]. Furthermore, intraperitoneal administration of IL-33 following wounding was shown to improve wound closure and collagen deposition, although angiogenesis was not assessed in this study [46]. IL-33 has been shown to induce the proliferation, migration, and morphologic differentiation of human endothelial cells *in vitro* [47], but its potential pro-angiogenic role during cutaneous wound healing remains to be determined. Thus, further study is needed on the role of IL-33 in wound healing.

Interleukin-1α is another member of the IL-1 family, but proIL-1α does not require caspase-1 activity for activation and can bind and activate the IL-1 receptor [14,48]. Interestingly, proIL-1α, -1β, -18 and-33 all lack a signal peptide and are secreted by an unconventional, endoplasmic reticulum/Golgi-independent pathway, which is not fully understood [39,49,50]. Activated macrophages from NLRP-3 or caspase-1 deficient mice not only release less IL-1β and IL-18, but also less IL-1α [28,44,45], establishing a possible role of caspase-1 and the inflammasome in unconventional protein secretion. To this extent, secretion of proIL-1α and another leaderless protein, FGF-2, was shown to be dependent on caspase-1 activity in activated macrophages and UV-irradiated keratinocytes [34]. Interleukin-1α is considered a less potent angiogenic factor compared to IL-1β, but may stimulate angiogenesis via the recruitment of inflammatory cells that are abundant sources of FGF and VEGF [51,52]. Thus, it is plausible that defective caspase-1-mediated protein secretion of these leaderless proteins in NLRP-3 KO and caspase-1 KO mice may contribute to impaired angiogenesis and wound healing. However, FGF-2 levels were not different in NLRP-3 KO mice versus wild type mice and were not altered with IL-1β treatment, thus, it is unlikely that the role of FGF-2 in wound healing is through the NLRP-3/caspase-1 pathway.

Our study is limited in that only one downstream NLRP-3 target (IL-1β) was investigated as a candidate to explain the impaired healing in NLRP-3 KO and caspase-1 KO mice. Although a lack of IL-1β appears to contribute to impaired early healing responses, other inflammasome targets may also contribute to the impaired healing phenotype in these mice. Another limitation is that the caspase-1 KO mice have been found to be deficient in caspase-11 [53,54], and so we cannot distinguish the role of these caspases in the impaired healing of wounds in caspase-1/11 KO mice. Nonetheless, because the impaired healing phenotype of NLRP-3 and caspase-1/11 KO mice were similar, our experiments indicate that the NLRP-3 inflammasome likely contributes to the early inflammatory phase of wound healing and that NLRP-3 signaling is important for efficient healing.

Collectively, findings from this study enhance our understanding of inflammation and wound healing and demonstrate the important role of NLRP-3 signaling in the early inflammatory response in tissue repair. Further studies elucidating the IL-1β-independent mechanism through which the NLRP-3/caspase-1 pathway mediates angiogenesis may reveal novel pathways involved in angiogenesis and wound healing.
